# Supplementary material for: Global evaluation of lineage-specific human papillomavirus capsid antigenicity using antibodies elicited by natural infection
Source: Nat Commun. 2024 Feb 21;15:1608. doi: 10.1038/s41467-024-45807-w (PMC10881982; doi:10.1038/s41467-024-45807-w)
Supplement: Supplementary file 1 — Supplementary Information [file 41467_2024_45807_MOESM1_ESM.pdf]

Supplementary Table 1. *Seropositivity rates by sample type, disease status, geographic region and lineage*

Genotype	Variable	Value	Total (N)	Seronegative (n)	Seropositive (n)	%	*p value*
HPV16	Sample	Plasma	343	197	146	43%	
	Sample	Serum	232	152	80	34%	*0.052*
	Disease	Disease	470	280	190	40%	
	Disease	No Disease	105	69	36	34%	*0.244*
	Region	Africa	320	187	133	42%	
	Region	Americas	136	80	56	41%	
	Region	Asia	113	82	31	27%	
	Region	Europe	6	0	6	100%	*0.001*
	Lineage	A	150	104	46	31%	
	Lineage	B	112	62	50	45%	
	Lineage	C	158	94	64	41%	
	Lineage	D	155	89	66	43%	*0.078*
HPV18	Sample	Plasma	220	167	53	24%	
	Sample	Serum	107	96	11	10%	*0.003*
	Disease	Disease	156	137	19	12%	
	Disease	No Disease	171	126	45	26%	*0.001*
	Region	Africa	89	73	16	18%	
	Region	Americas	158	119	39	25%	
	Region	Asia	78	69	9	12%	
	Region	Europe	2	2	0	0%	*0.092*
	Lineage	A	197	161	36	18%	
	Lineage	B	126	98	28	22%	
	Lineage	C	4	4	0	0%	*0.418*
HPV31	Sample	Plasma	255	161	94	37%	
	Sample	Serum	176	125	51	29%	*0.089*
	Disease	Disease	98	72	26	27%	
	Disease	No Disease	333	214	119	36%	*0.090*
	Region	Africa	40	24	16	40%	
	Region	Americas	357	239	118	33%	
	Region	Asia	23	16	7	30%	
	Region	Europe	11	7	4	36%	*0.820*
	Lineage	A	148	94	54	36%	
	Lineage	B	145	101	44	30%	
	Lineage	C	138	91	47	34%	*0.534*
HPV33	Sample	Plasma	134	75	59	44%	
	Sample	Serum	55	31	24	44%	*0.961*
	Disease	Disease	52	24	28	54%	
	Disease	No Disease	137	82	55	40%	*0.090*
	Region	Africa	13	6	7	54%	
	Region	Americas	141	80	61	43%	
	Region	Asia	23	14	9	39%	
	Region	Europe	12	6	6	50%	*0.816*
	Lineage	A	149	80	69	46%	
	Lineage	B	28	18	10	36%	
	Lineage	C	12	8	4	33%	*0.437*
HPV45	Sample	Plasma	180	149	31	17%	
	Sample	Serum	119	114	5	4%	*0.001*
	Disease	Disease	109	104	5	5%	
	Disease	No Disease	190	159	31	16%	*0.003*
	Region	Africa	55	48	7	13%	
	Region	Americas	200	171	29	14%	
	Region	Asia	42	42	0	0%	
	Region	Europe	2	2	0	0%	*0.066*
	Lineage	A	150	133	17	11%	
	Lineage	B	149	130	19	13%	*0.706*
HPV52	Sample	Plasma	197	98	99	50%	
	Sample	Serum	38	21	17	45%	*0.533*
	Disease	Disease	65	33	32	49%	
	Disease	No Disease	170	86	84	49%	*0.980*
	Region	Africa	25	12	13	52%	
	Region	Americas	155	81	74	48%	
	Region	Asia	52	24	28	54%	
	Region	Europe	3	2	1	33%	*0.811*
	Lineage	A	146	75	71	49%	
	Lineage	B	35	18	17	49%	
	Lineage	C	38	20	18	47%	
	Lineage	D	16	6	10	62%	*0.752*
HPV58	Sample	Plasma	162	97	65	40%	
	Sample	Serum	37	18	19	51%	*0.212*
	Disease	Disease	51	29	22	43%	
	Disease	No Disease	147	85	62	42%	*0.688*
	Region	Africa	33	17	16	48%	
	Region	Americas	123	70	53	43%	
	Region	Asia	42	27	15	36%	
	Region	Europe	1	1	0	0%	*0.567*
	Lineage	A	149	86	63	42%	
	Lineage	B	13	4	9	69%	
	Lineage	C	18	10	8	44%	
	Lineage	D	19	15	4	21%	*0.060*

N, total number of samples tested; *n*, resulting number of samples testing seropositive or seronegative; *p* value, χ^2 (Chi squared; 2-sided, exact *p* values) test for differences in proportions

Supplementary Table 2. *Lineage-specific geometric mean (95%CI) neutralization titers*

Genotype	Infection lineage	N	Metric	PsV-A	PsV-B	PsV-C	PsV-D
HPV16	A	46	n	42	43	44	43
			GMT (95%CI)	361 (233-560)	381 (253-572)	346 (233-514)	321 (216-478)
			p value		0.970	0.618	0.331
	B	50	n	44	46	44	40
			GMT (95%CI)	377 (216-657)	390 (227-671)	280 (163-482)	284 (160-505)
			p value	0.702		0.188	0.021
	C	64	n	50	50	64	50
			GMT (95%CI)	201 (133-305)	209 (136-321)	481 (320-724)	215 (140-329)
			p value	<0.001	<0.001		<0.001
	D	66	n	63	64	60	64
			GMT (95%CI)	318 (220-459)	335 (238-472)	284 (200-404)	330 (235-464)
			p value	0.837	0.636	0.483	
HPV18	A	36	n	36	35	35	
			GMT (95%CI)	275 (183-414)	271 (174-421)	228 (150-347)	
			p value		0.226	0.043	
	B	28	n	27	27	27	
			GMT (95%CI)	165 (125-216)	168 (130-216)	146 (114-188)	
			p value	0.802		0.001	
	C	0	n				
			GMT (95%CI)	N/A	N/A	N/A	
			p value				
HPV31	A	54	n	51	53	50	
			GMT (95%CI)	241 (180-324)	221 (172-285)	196 (148-260)	
			p value		0.115	0.024	
	B	44	n	40	42	42	
			GMT (95%CI)	176 (129-240)	181 (134-246)	188 (137-257)	
			p value	0.556		0.401	
	C	47	n	45	45	47	
			GMT (95%CI)	159 (125-203)	175 (137-224)	231 (182-294)	
			p value	<0.001	<0.001		
HPV33	A	69	n	66	49	38	
			GMT (95%CI)	381 (265-548)	132 (93-187)	89 (63-127)	
			p value		<0.001	<0.001	
	B	10	n	6	9	8	
			GMT (95%CI)	91 (34-245)	331 (119-923)	188 (65-543)	
			p value	0.017		0.032	
	C	4	n	3	3	3	
			GMT (95%CI)	163 (14-1864)	155 (14-1682)	150 (9-2644)	
			p value	0.465	0.853		
HPV45	A	17	n	17	14		
			GMT (95%CI)	180 (134-241)	110 (71-170)		
			p value		0.040		
	B	19	n	18	18		
			GMT (95%CI)	232 (135-398)	218 (133-357)		
			p value	0.520			
HPV52	A	71	n	68	64	69	50
			GMT (95%CI)	266 (202-352)	154 (122-194)	250 (196-318)	91 (70-118)
			p value		<0.001	0.006	<0.001
	B	17	n	16	13	16	10
			GMT (95%CI)	235 (124-443)	124 (67-228)	197 (110-355)	97 (44-213)
			p value	0.004		0.008	0.905
	C	18	n	18	16	18	11
			GMT (95%CI)	346 (189-629)	127 (69-235)	297 (172-510)	72 (42-123)
			p value	0.199	<0.001		<0.001
	D	10	n	7	7	7	9
			GMT (95%CI)	119 (47-301)	102 (44-240)	112 (45-276)	300 (127-705)
			p value	0.203	0.059	0.169	
HPV58	A	63	n	63	58	10	62
			GMT (95%CI)	386 (287-520)	270 (191-382)	32 (27-37)	376 (276-511)
			p value		0.203	<0.001	0.686
	B	9	n	6	7	4	6
			GMT (95%CI)	94 (32-282)	108 (36-329)	47 (26-85)	97 (31-304)
			p value	0.550		0.260	0.403
	C	8	n	7	6	6	5
			GMT (95%CI)	133 (64-275)	108 (46-254)	141 (52-379)	84 (36-197)
			p value	0.575	0.401		0.263
	D	4	n	2	2	2	2
			GMT (95%CI)	57 (8-384)	53 (13-214)	74 (10-544)	67 (8-559)
			p value	0.353	0.842	0.853	

N, number of sera positive for antibodies against any lineage antigen; *n*, number of sera positive for antibodies against indicated antigen; GMT, geometric mean (95%CI) neutralization titer; N/A, not applicable; *p* value, Wilcoxon paired rank test (2-sided, exact *p* values).

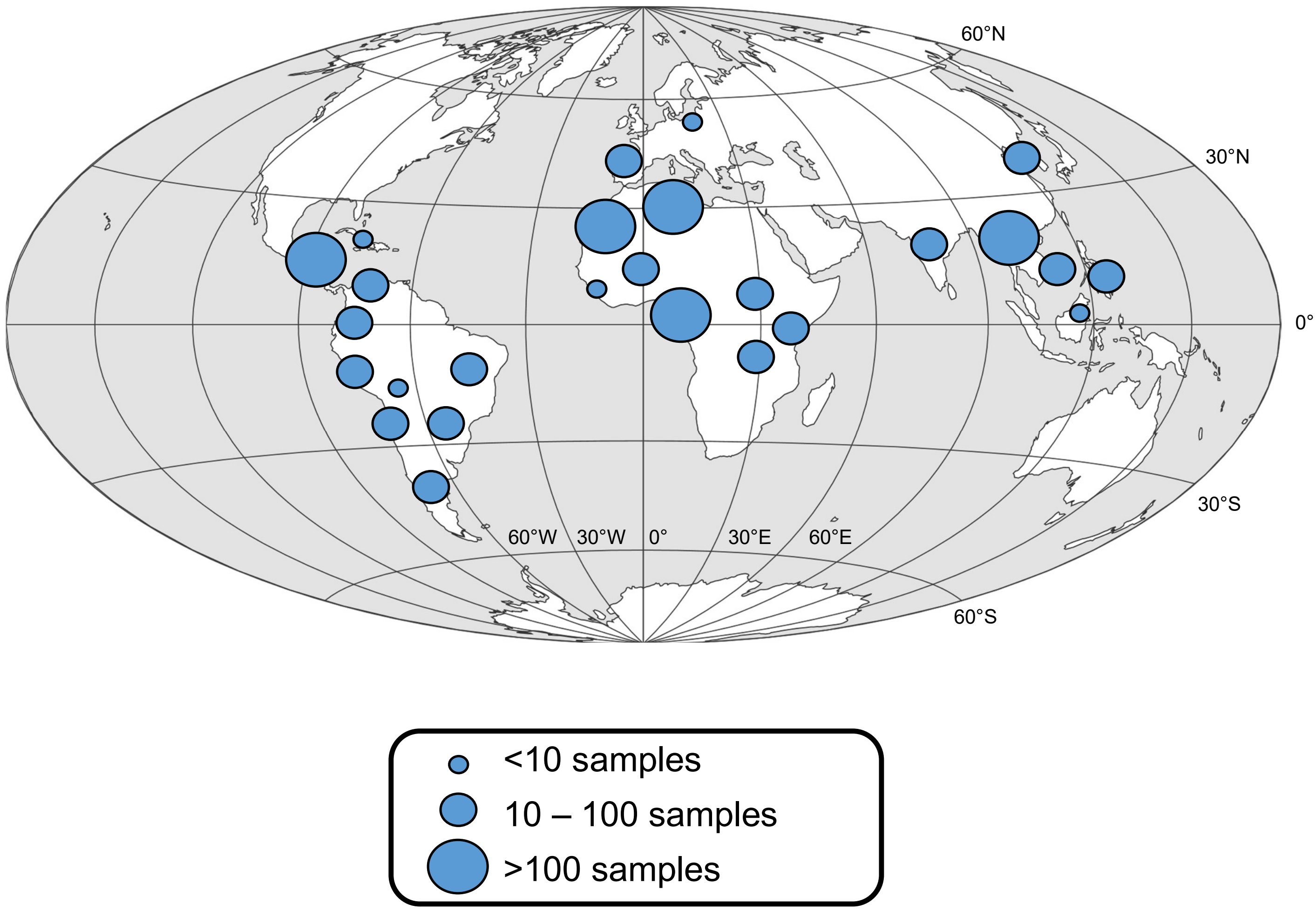

Region	Country	16A	16B	16C	16D	18A	18B	18C	31A	31B	31C	33A	33B	33C	45A	45B	52A	52B	52C	52D	58A	58B	58C	58D	Total
Africa	Algeria	21	15	23	17	13	1	1			5	1			4	4	1								106
Africa	Guinea		2	3									1		1										7
Africa	Kenya	2	6	31		3	12			1	1	2			8	3	2	1						3	75
Africa	Mali	4	24	15		4	9	3			1			1	9	2	2					4	2	1	81
Africa	Morocco	12	25	47	10	19	1		3		6	3			2	4	4					1			137
Africa	Nigeria	5	9	14		1	13		1		22	1	1	1	11	2	14				4	6	10	2	117
Africa	Tanzania		12	5			6					1			3		1								28
Africa	Uganda		12	5	1		3						1		2										24
Americas	Argentina	15		1	8	7			5	5	11	3				3	11		1		6				76
Americas	Bolivia				1		1		3	2	1				2	1	1								12
Americas	Brazil	5		3	24	12	1		7			6			2	5	1				9				75
Americas	Chile				5					5	1	5			1	3	3	1							24
Americas	Colombia					2			13	1		3									3				22
Americas	Costa Rica		4		9	47	74		76	121	82	82	25	10	69	102	87	5	15	12	81		5	12	918
Americas	Cuba		1		3		1		1							1	1								8
Americas	Panama		1	2	3		3		1		1				3	2	1			1	2				20
Americas	Paraguay				3				1		2	5			2	1	1					1			16
Americas	Peru	17			31	10			15	3		2			3		8		6		4				99
Asia	India	13			2	13					2	6			3	2		2			4				47
Asia	Indonesia														1	3									4
Asia	Korea	5				4			1			5				1		3		1	5				25
Asia	Philippines	10	1	2	5	27	1		4						23	5	3	8	1		9				99
Asia	Thailand	33		7	27	29			13	1		6				4	1	9	13	2	13	1		1	160
Asia	Vietnam	8				4			2			6					3	6			8		1		38
Europe	Poland									1	1														2
Europe	Spain				6	2			2	5	2	12			1	1	1		2		1				35
	Total	150	112	158	155	197	126	4	148	145	138	149	28	12	150	149	146	35	38	16	149	13	18	19	2255

Supplementary Figure 1 *Geographical distribution of samples included in this study*

Top panel, world map with 30° indicative latitude and longitude grid (Tentotwo; 1:110m Natural Earth Datasets CC BY-SA 3.0; https://commons.wikimedia.org/w/index.php?curid=21507855) highlighting geographical dispersal and approximate number of samples included within this collection. Bottom panel, numbers of samples representing each region, country, type and lineage in this study, where each cell contains *n* number of samples with green intensity reflecting the proportion within the collection.

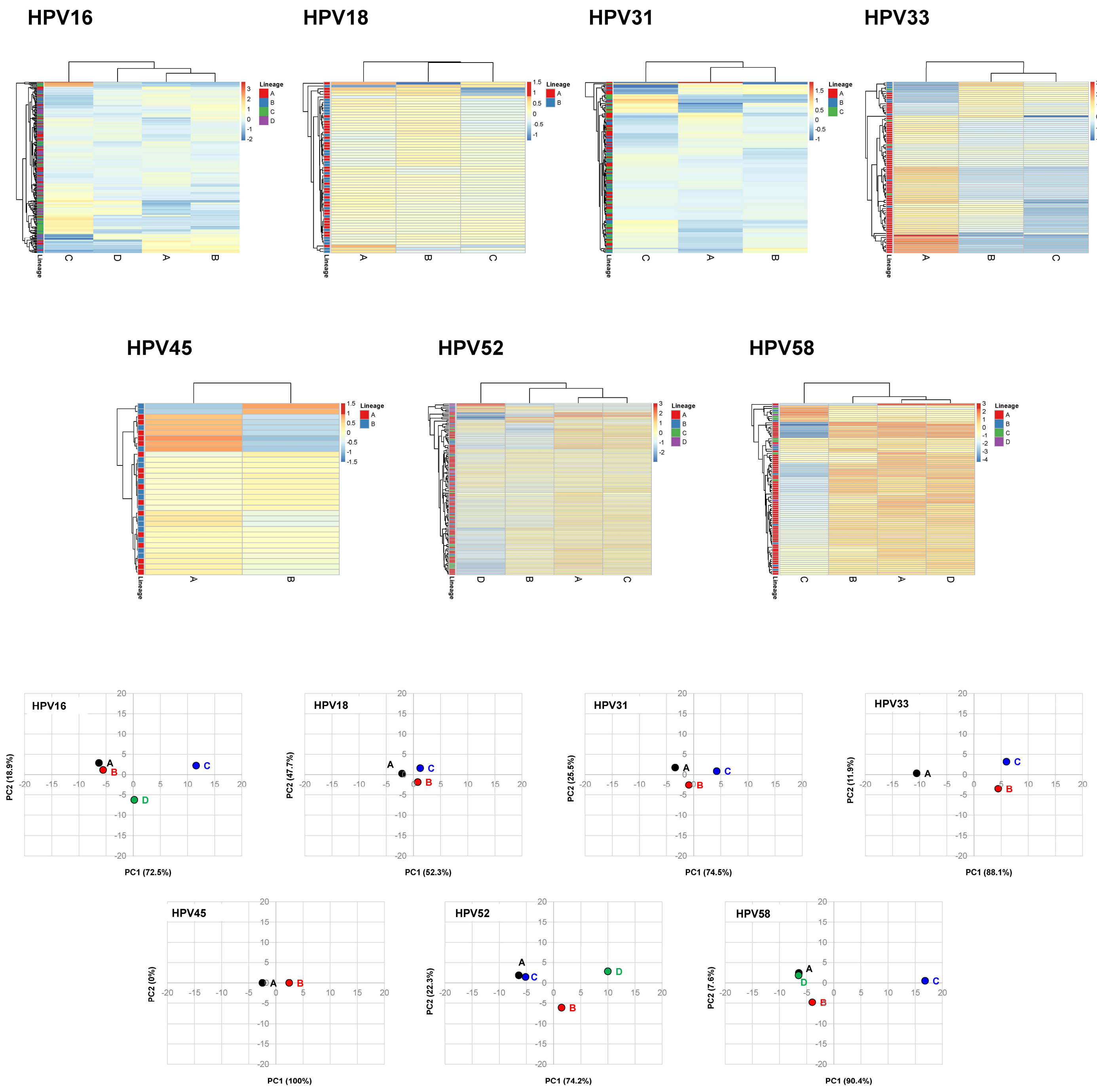

Supplementary Figure 2 *Use of additional antigen clustering tools*

Top panel, hierarchical clustering and heatmap using indicated relative scale. Natural log neutralizing antibody titers were reordered according to serological and antigen dendrograms constructed from the resulting Euclidean distance matrices. Serum side bar denotes natural infection lineage from which serum (or plasma) sample derived according to key. Bottom panel, principal component analysis to define relative antigen position in two-dimensional space: lineage A (black), B (red), C (blue) and D (green). The PC1 and PC2 channels accounted for the majority of the variance across these datasets as indicated: HPV16 (91%), HPV18 (100%), HPV31 (100%), HPV33 (100%), HPV45 (100%), HPV52 (97%), HPV58 (98%). Both analyses made use of the ClustVis web tool (https://biit.cs.ut.ee/clustvis/).

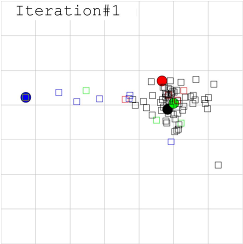

Fold	A	B	C	D
A		1.8	17.4	1.2
B			15.7	1.7
C				19.5

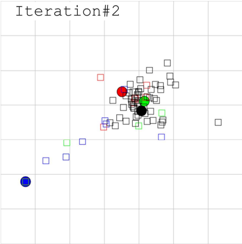

Fold	A	B	C	D
A		1.7	15.2	1.2
B			14.0	1.6
C				17.7

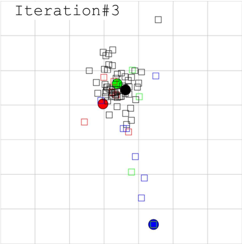

Fold	A	B	C	D
A		1.7	15.6	1.2
B			13.6	1.6
C				18.2

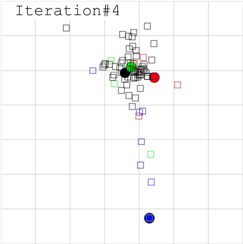

Fold	A	B	C	D
A		1.8	19.1	1.2
B			16.7	1.7
C				21.1

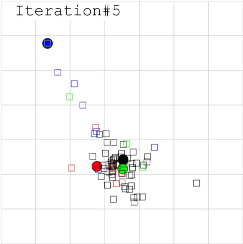

Fold	A	B	C	D
A		1.7	16.1	1.2
B			14.2	1.7
C				18.7

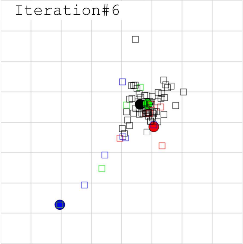

Fold	A	B	C	D
A		1.8	19.0	1.2
B			16.5	1.7
C				21.3

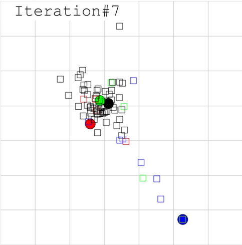

Fold	A	B	C	D
A		1.7	15.7	1.2
B			14.4	1.6
C				18.1

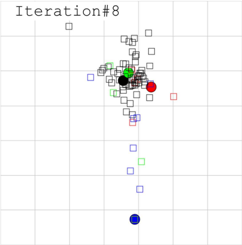

Fold	A	B	C	D
A		1.8	16.1	1.2
B			14.3	1.7
C				18.6

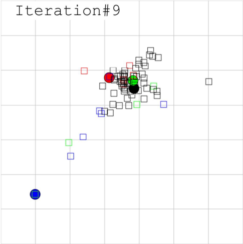

Fold	A	B	C	D
A		1.7	18.1	1.2
B			15.8	1.6
C				20.1

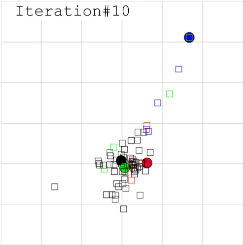

Fold	A	B	C	D
A		1.7	18.6	1.2
B			15.7	1.6
C				20.5

Mean (95%CI)	A	B	C	D
A		1.7<br>1.7 - 1.8	17.0<br>16.1 - 18.0	1.2<br>1.2 - 1.2
B			15.0<br>14.3 - 15.7	1.7<br>1.6 - 1.7
C				19.3<br>18.5 - 20.2

Full dataset

	Fold	B	C	D
16	A	1.2	2.7	1.9
	B		2.6	1.7
	C			2.0
18	A	1.3	1.4	
	B		1.3	
31	A	1.3	1.6	
	B		1.4	
33	A	4.2	5.9	
	B		1.7	
52	A	2.3	1.1	3.9
	B		2.2	3.5
	C			3.6
58	A	1.7	17.2	1.2
	B		15.7	1.7
	C			19.8

Mean iterations (n=10)

	Fold	B	C	D
16	A	1.2	2.7	2.0
	B		2.6	1.7
	C			2.0
18	A	1.3	1.4	
	B		1.3	
31	A	1.3	1.6	
	B		1.4	
33	A	4.2	5.9	
	B		1.7	
52	A	2.3	1.1	3.8
	B		2.2	3.4
	C			3.6
58	A	1.7	17.0	1.2
	B		15.0	1.7
	C			19.3

Supplementary Figure 3 *Resampling of 90% of neutralization data for antigenic cartography iterations*

To test the robustness of the antigenic mapping for each genotype a 10% data redaction method was employed. The dataset for each genotype was subjected to 10 such randomizations (iterations) creating 10 pseudo-replicate antigenic maps. An example using HPV58 is shown with lineages coded as follows: A, black; B, red; C, blue; D, green. Antigenic distances between antigens were summarized as the mean (95%CI). Comparisons of mean distances for the full dataset and the iterative dataset are summarized. Fold difference between indicated antigens shown with <2-fold (green), 2-4 fold (gold) and >4 fold (red) differences highlighted as indicated. Analysis restricted to genotypes with >2 lineages for comparability, thus does not include HPV45.

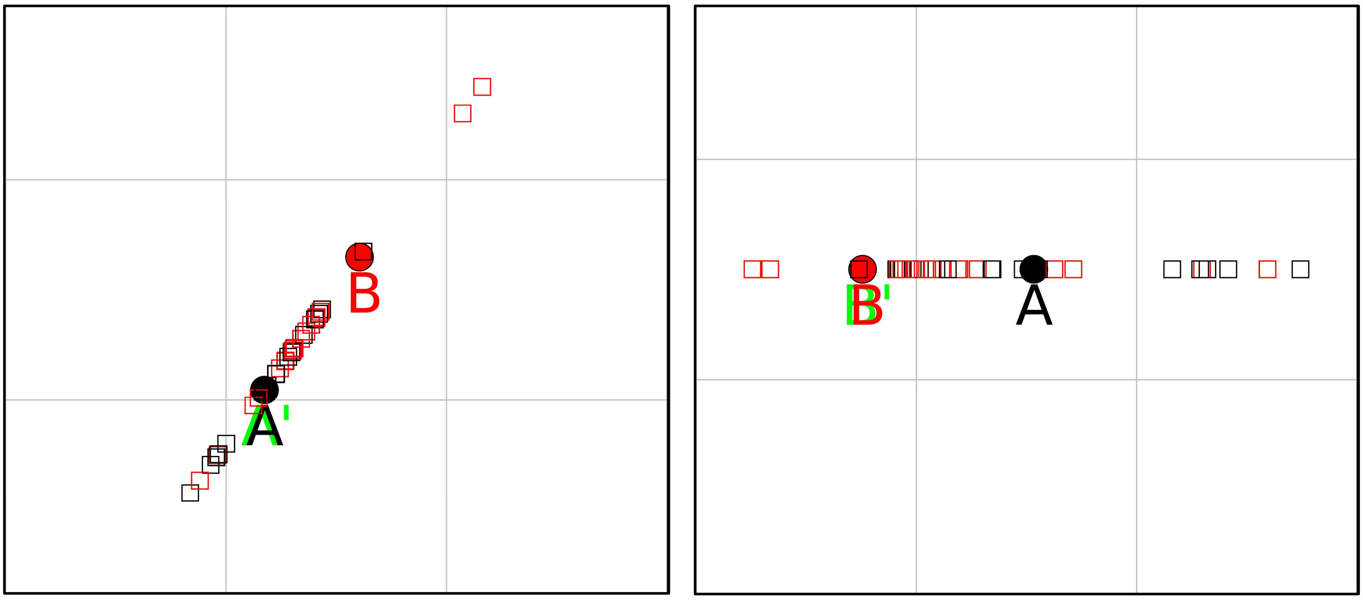

Supplementary Figure 4 *Antigenic mapping of HPV45 lineage A and B pseudovirus antigens*

To estimate the antigenic distance between lineage antigens A and B, a dummy dataset was included in each map based upon a copy of lineage A (A', left panel) or lineage B data (B', right panel). Lineage antigens A (black), B (red) and A'/B' copy (green) as indicated.

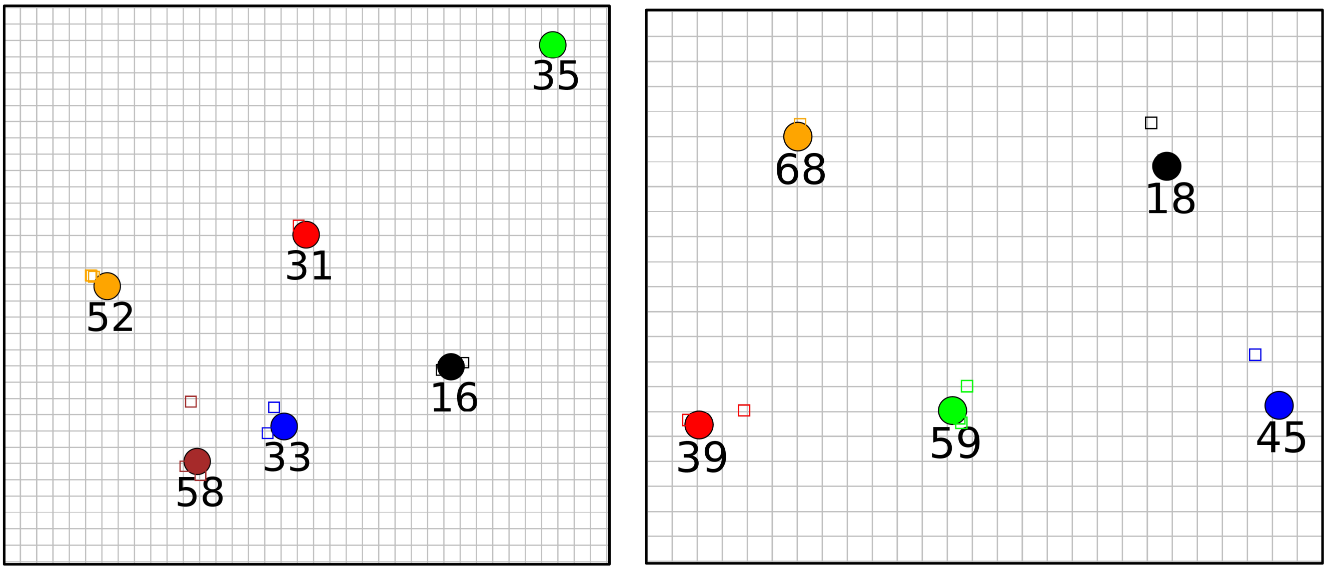

Supplementary Figure 5 *Antigenic mapping of type-specific rabbit neutralizing antibody responses*

Left panel, Alpha-9 types (HPV16, 18, 31, 33, 35, 52 and 58) and right panel, Alpha-7 types (HPV18, 39, 45, 59 and 68). Filled circles and open squares represent type-specific pseudovirus antigens and rabbit sera (n=3 per type), respectively, and are similarly colored for each type. In each antigenic map the grey grid squares represent 1 antigenic unit (AU), which is equivalent to a 2-fold distance; thus, three grid squares is equivalent to an 8-fold (2^3) distance.

Genotype	Total	Single	Dual	Multiple
HPV16	575	541	31	3
HPV18	327	295	32	0
HPV31	431	392	38	1
HPV33	189	169	16	4
HPV45	299	279	18	2
HPV52	235	204	27	4
HPV58	199	165	30	4
Total	2255	2045	192	18
%	100%	90.7%	8.5%	0.8%

Full dataset

	Fold	B	C	D
16	A	1.2	2.7	1.9
	B		2.6	1.7
	C			2.0
18	A	1.3	1.4	
	B		1.3	
31	A	1.3	1.6	
	B		1.4	
33	A	4.2	5.9	
	B		1.7	
52	A	2.3	1.1	3.9
	B		2.2	3.5
	C			3.6
58	A	1.7	17.2	1.2
	B		15.7	1.7
	C			19.8

Single infections only

	Fold	B	C	D
16	A	1.2	2.7	2.0
	B		2.6	1.7
	C			2.0
18	A	1.3	1.4	
	B		1.3	
31	A	1.3	1.6	
	B		1.4	
33	A	4.4	6.3	
	B		1.8	
52	A	2.4	1.1	4.0
	B		2.4	3.8
	C			3.7
58	A	1.7	18.8	1.2
	B		18.1	1.6
	C			21.6

Supplementary Figure 6 *Assessment of antigenic distance using single vs. mixed infection samples*

Top panel, a small number of DNA samples exhibited mixed infections. Additional coded serum samples were included to account for this multiplicity. Bottom panel, a subset analysis was conducted where the antigenic distances were estimated for those samples derived from single infections only and a comparison made with those that included all samples. Fold difference between indicated antigens shown with <2-fold (green), 2-4 fold (gold) and >4 fold (red) differences highlighted as indicated. Analysis was restricted to genotypes with >2 lineages for comparability, thus does not include HPV45.

Impact analysis	Samples (n)	Fold	B	C	D
Original dataset	*n=226 seropositive samples*	A	1.2	2.7	1.9
		B		2.6	1.7
		C			2.0
Impact of geographical origin on antigenic distance estimates	*Asia and the Americas (n=87; no samples from Africa or Europe)*	A	1.3	2.3	1.9
		B		2.0	1.4
		C			1.6
	Africa and Asia (n=164; no samples from the Americas or Europe)	A	1.1	2.8	1.9
		B		2.8	1.8
		C			2.2
	Africa and the Americas (n=189; no samples from Asia or Europe)	A	1.3	2.9	2.1
		B		2.7	1.7
		C			2.1
	Africa only (n=133)	A	1.1	3.1	2.0
		B		3.0	1.9
		C			2.4
	The Americas only (n=56)	A	1.4	2.5	2.0
		B		2.1	1.4
		C			1.7
	Asia and Europe only (n=37)	A	1.2	2.0	1.5
		B		1.8	1.6
		C			1.4
Impact of seropositivity rate on antigenic distance estimates	*Reduced lineage A samples (n=27/46; n=207 total samples)*	A	1.2	2.6	1.9
		B		2.6	1.6
		C			2.0
	Reduced lineage B samples (n=19/50; n=195 total samples)	A	1.2	2.6	2.0
		B		2.5	1.6
		C			1.9
	Reduced lineage C samples (n=32/64; n=194 total samples)	A	1.3	2.5	1.9
		B		2.4	1.6
		C			1.8
	Reduced lineage D samples (n=30/66; n=190 total samples)	A	1.2	2.7	2.0
		B		2.6	1.7
		C			2.1

Supplementary Figure 7 *Impact assessment of geographic origin and seropositivity rate on antigenic distance*

Estimates of antigenic distance between HPV16 lineage antigens following inclusion of samples from indicated geographical regions and following removal of *ca*. 50% of seropositive samples to simulate a lower seropositivity rate for the indicated lineages compared to the original dataset. Fold difference between indicated antigens shown with <2-fold (green), 2-4 fold (gold) and >4 fold (red) differences highlighted as indicated.

Method	Type	N	Fold	B	C	D
Original dataset	HPV33	A=69 B=10 C=4	A	4.2	5.9	
			B		1.7	
			C			
	HPV52	A=71 B=17 C=18 D=10	A	2.3	1.1	3.9
			B		2.2	3.5
			C			3.6
	HPV58	A=63 B=9 C=8 D=4	A	1.7	17.2	1.2
			B		15.7	1.7
			C			19.8
Proportionate randomized resampling with replacement	HPV33	A=127 B=17 C=7	A	4.1 (3.8 – 4.4)	5.9 (5.5 – 6.3)	
			B		1.8 (1.7 – 1.8)	
			C			
	HPV52	A=125 B=28 C=29 D=18	A	2.2 (2.2 – 2.3)	1.1 (1.0 – 1.1)	3.8 (3.6 – 3.9)
			B		2.2 (2.1 – 2.3)	3.6 (3.4 – 3.8)
			C			3.6 (3.5 – 3.7)
	HPV58	A=147 B=23 C=20 D=10	A	1.7 (1.6 – 1.8)	16.2 (14.7 – 17.7)	1.2 (1.2 – 1.3)
			B		14.5 (12.8 – 16.2)	1.7 (1.6 – 1.8)
			C			18.9 (17.0 – 20.9)
Disproportionate randomized resampling with replacement	HPV33	A=50 B=50 C=50	A	4.3 (4.1 – 4.5)	4.8 (4.6 – 5.0)	
			B		1.5 (1.4 – 1.5)	
			C			
	HPV52	A=50 B=50 C=50 D=50	A	2.3 (2.3 – 2.4)	1.1 (1.0 – 1.1)	4.1 (3.9 – 4.3)
			B		2.3 (2.2 – 2.4)	4.0 (3.8 – 4.3)
			C			3.9 (3.8 – 4.1)
	HPV58	A=50 B=50 C=50 D=50	A	1.8 (1.8 – 1.9)	8.1 (7.6 – 8.6)	1.3 (1.3 – 1.4)
			B		6.2 (5.9 – 6.5)	1.8 (1.7 – 1.9)
			C			9.5 (8.9 – 10.2)

Supplementary Figure 8 *Antigenic distance estimates following randomized resampling with replacement*

Estimates of antigenic distance (mean, 95%CI) between lineage antigens following two randomized resampling with replacement evaluations compared to the original dataset. The proportionate randomized resampling with replacement randomly selected samples from the type-specific dataset where N represents the mean number of samples for each lineage after 10 iterations and is expected to maintain the structure of the original dataset. The disproportionate randomized resampling with replacement randomly selected samples until a target of N=50 samples for each lineage was reached and therefore is not expected to retain the structure of the original dataset. Fold difference between indicated antigens shown with <2-fold (green), 2-4 fold (gold) and >4 fold (red) differences highlighted as indicated.
