# Supplementary material for: Global evaluation of lineage-specific human papillomavirus capsid antigenicity using antibodies elicited by natural infection
Source: Nat Commun. 2024 Feb 21;15:1608. doi: 10.1038/s41467-024-45807-w (PMC10881982; doi:10.1038/s41467-024-45807-w)
Supplement: Supplementary file 3 — Reporting Summary [file 41467_2024_45807_MOESM3_ESM.pdf]

Corresponding author(s): Gathoni Kamuyu, Filomeno Coelho da Silva, Vanessa Tenet, John Schussler, Anna Godi, Rolando Herrero, Carolina Porras, Lisa Mirabello, John T. Schiller, Mónica S. Sierra, Aimée R. Kreimer, Gary M. Clifford, and Simon Beddows

Last updated by author(s): Jan 30, 2024

Reporting Summary

Nature Portfolio wishes to improve the reproducibility of the work that we publish. This form provides structure for consistency and transparency in reporting. For further information on Nature Portfolio policies, see our Editorial Policies and the Editorial Policy Checklist.

Statistics

For all statistical analyses, confirm that the following items are present in the figure legend, table legend, main text, or Methods section.

n/a	Confirmed	
☐	☒	The exact sample size (*n*) for each experimental group/condition, given as a discrete number and unit of measurement
☐	☒	A statement on whether measurements were taken from distinct samples or whether the same sample was measured repeatedly
☐	☒	The statistical test(s) used AND whether they are one- or two-sided<br>*Only common tests should be described solely by name; describe more complex techniques in the Methods section.*
☐	☒	A description of all covariates tested
☐	☒	A description of any assumptions or corrections, such as tests of normality and adjustment for multiple comparisons
☐	☒	A full description of the statistical parameters including central tendency (e.g. means) or other basic estimates (e.g. regression coefficient) AND variation (e.g. standard deviation) or associated estimates of uncertainty (e.g. confidence intervals)
☒	☐	For null hypothesis testing, the test statistic (e.g. *F*, *t*, *r*) with confidence intervals, effect sizes, degrees of freedom and *P* value noted<br>*Give P values as exact values whenever suitable.*
☒	☐	For Bayesian analysis, information on the choice of priors and Markov chain Monte Carlo settings
☒	☐	For hierarchical and complex designs, identification of the appropriate level for tests and full reporting of outcomes
☐	☒	Estimates of effect sizes (e.g. Cohen's *d*, Pearson's *r*), indicating how they were calculated

Our web collection on statistics for biologists contains articles on many of the points above.

Software and code

Policy information about availability of computer code

Data collection	Data collection was carried out using Excel and Access software and made use of Visual Basic for Applications macros to improve and reduce the risk of data handling steps.
Data analysis	Data analysis was carried out using: Stata 17.0 SE (StataCorp); sample size estimate (https://statulator.com/SampleSize/ss2PM.html); hierarchical clustering (https://www.hiv.lanl.gov/content/sequence/HEATMAP/heatmap.html); antigenic cartography (https://acmacs-web.antigenic-cartography.org/); GraphPad Prism v9.1.1; Molecular Evolutionary Genetics Analysis (MEGA) v7.0.26; crystal mapping (https://www.rcsb.org/; Swiss-PdbViewer v4.1 (https://spdbv.unil.ch/)); additional clustering tools ClustVis (https://biit.cs.ut.ee/clustvis/)

For manuscripts utilizing custom algorithms or software that are central to the research but not yet described in published literature, software must be made available to editors and reviewers. We strongly encourage code deposition in a community repository (e.g. GitHub). See the Nature Portfolio guidelines for submitting code & software for further information.

Data

Policy information about availability of data

All manuscripts must include a data availability statement. This statement should provide the following information, where applicable:

- Accession codes, unique identifiers, or web links for publicly available datasets
- A description of any restrictions on data availability
- For clinical datasets or third party data, please ensure that the statement adheres to our policy

See Data availability section in manuscript text:

The data that support the findings of this study are available upon request from the IARC (Gary M. Clifford) and the NCI (Aimée R. Kreimer). The data are not publicly available as restrictions apply to the availability of these data, which were used under license for the current study. Data are, however, available upon reasonable request made to the indicated author.

De-identified participant data from the Costa Rica Vaccine Trial (CVT) can be shared with outside collaborators for research to understand more about the performance of the HPV vaccine, immune response to the vaccine, and broader study factors associated with the natural history of HPV infection and risk factors for infection and disease. Outside collaborators can apply to access the protocols and data online. For the trial summary, current publications, and contact information for data access see: Human Papillomavirus (HPV) Vaccine Trial in Costa Rica (CVT) - National Cancer Institute. For the Guanacaste Natural History Study (NHS) summary and contact information for data access see: https://dceg.cancer.gov/research/cancer-types/cervix/guanacaste-hpv-natural-history.

The pentamer crystal structures of HPV33 (Protein Data Bank [PDB; https://www.wwpdb.org/ and https://www.rcsb.org/] accession number: 6IGE.2; https://doi.org/10.2210/pdb6IGE/pdb), HPV52 (6IGF.1; https://doi.org/10.2210/pdb6IGF/pdb) and HPV58 (5Y9E.1; https://doi.org/10.2210/pdb5Y9E/pdb) were used to highlight lineage-specific amino acid polymorphisms.

Research involving human participants, their data, or biological material

Policy information about studies with human participants or human data. See also policy information about sex, gender (identity/presentation), and sexual orientation and race, ethnicity and racism.

Reporting on sex and gender	The number of samples from females (self-reported) representing each group (genotype, lineage) is given in the Supplementary Material and summarized in the manuscript results section.
Reporting on race, ethnicity, or other socially relevant groupings	No reporting of race, ethnicity or other socially relevant groupings was made in this report.
Population characteristics	Samples were included if the individual had an assigned lineage infection status. Covariates included sample type (serum or plasma); disease status; geographic origin and infecting genotype and lineage.
Recruitment	Samples were assembled from existing archives.
Ethics oversight	Samples were assembled from existing archives wherein samples were collected from ethically approved studies (references cited in the manuscript) with written, informed consent. Ethical approval for the use for these samples for the current study was made following approval from local institutional (International Agency for Research on Cancer and National Cancer Institute) review boards.

Note that full information on the approval of the study protocol must also be provided in the manuscript.

Field-specific reporting

Please select the one below that is the best fit for your research. If you are not sure, read the appropriate sections before making your selection.

☒ Life sciences ☐ Behavioural & social sciences ☐ Ecological, evolutionary & environmental sciences

For a reference copy of the document with all sections, see nature.com/documents/nr-reporting-summary-flat.pdf

Life sciences study design

All studies must disclose on these points even when the disclosure is negative.

Sample size	Sample size was determined a priori as 150 samples per lineage using published method (Dell RB, Holleran S, Ramakrishnan R. 2002. Sample size determination. Ilar j 43:207-213), corroborated by an estimate based upon effect size (Cohen's d; https://statulator.com/SampleSize/ss2PM.html) and anticipated seropositivity rates estimated from the literature (see Methods section of manuscript).
Data exclusions	No data were excluded.
Replication	Each sample was titrated once for the primary dataset but a subset of seropositive samples (mean 4%; range 1 – 7% of total samples depending on genotype; n=101 total samples) was subjected to repeat testing for quality assurance purposes, resulting in a median Log10

titer ratio of 0.98 (inter-quartile range, IQR, 0.94 – 1.01) and a Pearson's correlation coefficient (r2) of 0.923 for the initial and repeat tests. These metrics support successful sample repeatability. To test the robustness of the antigenic distance estimates, resampling iterations (without replacement) for all types were carried out wherein 10 pseudo-replicate antigenic maps containing 90% of data (following a random 10% redaction) were constructed and the Mean (95%CI) antigenic distances were compared to those derived using the full dataset. These iterative estimates were close to those derived using the full dataset. In addition, distance estimates were made following removal of ca. 50% of the seropositive samples for each lineage of HPV16 and two sampling (with replacement) evaluations were made for HPV33, HPV52 and HPV58. In all cases the distance outcomes were similar to those derived using the original dataset.

Randomization: Observational study, no randomization was done.

Blinding: The testing laboratory received coded serum (or plasma) samples representing the infecting genotype for that individual but was blinded to the associated metadata including assignment of natural infection lineage, sample type, disease state and geographical region until testing was complete.

Reporting for specific materials, systems and methods

We require information from authors about some types of materials, experimental systems and methods used in many studies. Here, indicate whether each material, system or method listed is relevant to your study. If you are not sure if a list item applies to your research, read the appropriate section before selecting a response.

Materials & experimental systems

n/a	Involved in the study	
☒	☐	Antibodies
☐	☒	Eukaryotic cell lines
☒	☐	Palaeontology and archaeology
☒	☐	Animals and other organisms
☒	☐	Clinical data
☒	☐	Dual use research of concern
☒	☐	Plants

Methods

n/a	Involved in the study	
☒	☐	ChIP-seq
☒	☐	Flow cytometry
☒	☐	MRI-based neuroimaging

Eukaryotic cell lines

Policy information about cell lines and Sex and Gender in Research

Cell line source(s): 293TT (https://www.atcc.org/products/crl-3467) cell line exhibiting epithelial morphology that was isolated from a human kidney. It has applications for production of papillomavirus and polyomavirus-based reporter vectors

Authentication: No formal authentication carried out. Cell line maintained under hygromycin B selection to promote maintenance of T antigen expression. Pseudovirus stocks assigned a TCID50 using 293TT cells to normalize the amount of input used for neutralization assay. Cell line limited to specific number of passages supported by evidence of appropriate assay performance.

Mycoplasma contamination: Cells were not tested for mycoplasma contamination.

Commonly misidentified lines (See ICLAC register): Commonly used misidentified cell lines were not used in this study.
